# Whole mitochondrial and chloroplast genome sequencing of Tunisian date palm cultivars: diversity and evolutionary relationships

**DOI:** 10.1186/s12864-023-09872-7

**Published:** 2023-12-13

**Authors:** Hammadi Hamza, Sara Villa, Sara Torre, Alexis Marchesini, Mohamed Ali Benabderrahim, Mokhtar Rejili, Federico Sebastiani

**Affiliations:** 1 Arid and Oases Cropping Laboratory, Arid Regions Institute, Route du Djorf, Medenine, 4119 Tunisia; 2 Institute for Sustainable Plant Protection, National Research Council of Italy (CNR), via Madonna del Piano 10, Sesto Fiorentino, Florence, 50019 Italy; 3 Present Address: Research Institute on Terrestrial Ecosystems (IRET), National Research Council of Italy (CNR), via Marconi 2, Porano, Terni, 05010 Italy; 4 NBFC, National Biodiversity Future Center, Piazza Marina 61, Palermo, 90133 Italy; 5 Laboratory of Biodiversity and Valorization of Arid Areas Bioresources (BVBAA) – Faculty of Sciences of Gabes, University of Gabes, Erriadh, Gabes, 6072 Tunisia; 6 Department of Biology, College of Sciences, Al Imam Mohammad Ibn Saud Islamic University (IMSIU), Riyadh, 11623 Saudi Arabia

**Keywords:** Cultivars, Intra-specific genetic diversity, Organellar genome sequencing, Phylogenetic relationships, *Phoenix dactylifera* L., Single nucleotide polymorphisms

## Abstract

**Background:**

Date palm (*Phoenix dactylifera* L.) is the most widespread crop in arid and semi-arid regions and has great traditional and socioeconomic importance, with its fruit well-known for its high nutritional and health value. However, the genetic variation of date palm cultivars is often neglected. The advent of high-throughput sequencing has made possible the resequencing of whole organelle (mitochondria and chloroplast) genomes to explore the genetic diversity and phylogenetic relationships of cultivated plants with unprecedented detail.

**Results:**

Whole organelle genomes of 171 Tunisian accessions (135 females and 36 males) were sequenced. Targeted bioinformatics pipelines were used to identify date palm haplotypes and genome variants, aiming to provide variant annotation and investigate patterns of evolutionary relationship. Our results revealed the existence of unique haplotypes, identified by 45 chloroplastic and 156 mitochondrial SNPs. Estimation of the effect of these SNPs on genes functions was predicted *in silico*.

**Conclusions:**

The results of this study have important implications, in the light of ongoing environmental changes, for the conservation and sustainable use of the genetic resources of date palm cultivars in Tunisia, where monoculture threatens biodiversity leading to genetic erosion. These data will be useful for breeding and genetic improvement programs of the date palm through selective cross-breeding.

**Supplementary Information:**

The online version contains supplementary material available at 10.1186/s12864-023-09872-7.

## Background

Date palm (*Phoenix dactylifera* L.) belongs to the Arecaceae family and is the most widely grown crop in arid and semi-arid regions, contributing to feed the population of several countries [1]. It is cultivated especially across North Africa, the Middle East, Arabian Gulf and western parts of South Asia [2, 3], with a number of known cultivars ranging between 2,000 and 5,000 [4], for a global production of date fruits amounting to about 9.8 million tons in 2021 [5]. Due to its high nutritional values, many agri-food technologies use date fruit byproducts as food ingredients as well [6]. This species is one of the earliest domesticated fruit tree crops, with archaeological findings from the Persian Gulf showing evidence of cultivation dating back to around 6,000 years ago [7, 8]; however, in spite of many years of research in a variety of disciplines, the details of its domestication history and diversification has remained a question without a definite answer [2].

In the southern Tunisia, the total number of date palm trees growing is about 6 million, with more than 200 cultivars represented. Such genetic heritage is the result of centuries of artificial and natural selection processes involving domestication, migration, breeding and environmental effects [9]. Consequently, date palm cultivars diverged developing specific agronomic traits, among which the most prevalent are fruit yield and quality. Nevertheless, national and international market pressure has driven date palm cultivation in Tunisia towards monoculture, with many cultivars that become unfamiliar even to the rural population: this threatens date palm biodiversity and can potentially lead to genetic erosion and loss of valuable genetic resources. In the light of ongoing climate change and other potential pressures (e.g. pathogens and pests), adaptation measures for crops, e.g. the selection and use of varieties with different environmental (or diseases) tolerances, are becoming increasingly essential and therefore the preservation of germplasm diversity is an urgent priority [10].

The date palm is a dioecious species, i.e. with naturally cross-pollination between male and female trees. Artificial pollination is required in commercial date production for ensuring good fruit yields and quality [11]. Date palms can be propagated either from seeds or vegetative offshoots. However, seed propagation implies that half of the progeny are males and there are currently no effective methods for sex determination of young date palm seedlings: it is therefore unsuitable for commercial production, which relies on offshoots planting. Palms are long-living plants and both seeds and vegetative offshoots are characterized by an extremely slow growth, with female palms starting to produce fruits only after 5–10 years, depending to the cultivar [12]. Furthermore, female cultivars have been so far identified based on fruit morphology and given local names mostly according to geographical origin, with the result of potentially different names corresponding to the same genotype. On the other hand, since variety/cultivar information is closely linked to the marketing of the fruit produced, this is traditionally neglected for male plants. All the above-mentioned peculiar features make genetic improvement (e.g. to enhance fruit production) difficult with the use of classical breeding techniques [13]. In angiosperms, both chloroplasts and mitochondria genomes are usually inherited from maternal parent, leading to a non-Mendelian inheritance pattern [14]. While chloroplast genomes are highly conserved, generally organized into circular DNA molecules varying between 120 and 160 kb in length [15, 16], plant mitochondrial genomes are highly variable in terms of both length and gene content [17]. The full-length organelle genomes sequencing can unveil DNA markers for cultivars discrimination and may allow the identification of protein-coding regions potentially involved in agronomically important traits. Within the species of agronomic interest, the date palm was among the first whose complete mitochondria and chloroplast genomes were sequenced and published (715,001 bp and 158,462 bp, respectively) [18–20]. The release of reference genomes enables the identification of Single Nucleotide Polymorphisms (SNPs) markers, which in turn can be used for assessing whole-genome diversity and genetic relationships between cultivars. Whole organellar genomes have been used in a great variety of studies to investigate genetic differentiation and evolution of plant species [21, 22]. Their widespread use could be attributed to their usual uniparental inheritance, high copy-number, and low frequency of recombination [23, 24]. Over the past decade, there have been numerous efforts to use molecular markers to characterize date palm biodiversity. However, most of these studies have been based on a limited set of traditional genetic markers (i.e. RAPD, SSR and AFLP or short chloroplast regions) [25, 26], including the so far performed genetic surveys focusing on Tunisian cultivars [27, 28]. Novel genomic approaches, allowing an in-depth knowledge and functional characterization of the genome, have proven to outperform traditional markers in assisted selection for plant breeding, also considering that many agronomically important traits are complex and affected by multiple genes [29, 30]. The advent of next-generation sequencing (NGS) has made possible the resequencing of whole genomes to explore the genetic diversity of plants. Among various NGS technologies, the use of rapid and cost-effective genome-skimming strategy, which consists of sequencing the genomic DNA of an individual at low nuclear genome coverage, has become increasingly popular in plant phylogenomics [21, 31–33]. This approach, capable of generating high-copy fractions of total genomic DNA (including organelle genomes, nuclear ribosomal DNA and other multi-copy elements), was chosen for our study because it facilitates the creation of extensive phylogenetic datasets at relatively low cost and without the need for massive bioinformatics resources. To date, little research has been carried out based on whole genome sequencing (WGS) of date palm organelle genomes. The intraspecific diversity of Tunisian date palm cultivars was previously investigated by using nuclear and organelle markers [34–37], but variation at whole genome sequence remained largely unexplored. The phylogenetic relationships among nine Saudi Arabian cultivars were analyzed in 2014 [38] by means of organelle genome re-sequencing. Mohamoud et al. [3] used organelle WGS in a broad scale study across the main date palm growing regions (North Africa, the Middle East, Arabian Gulf and western parts of South Asia) to investigate the history and origin of date palm cultivation through variation in organelle genomes of different cultivars. Specifically, the latter study used only five Tunisian cultivars (i.e. Allig, Hamrata, Grenja, Nefred and Deglet Nour) and found that they could be divided into two neighboring haplotypes: NA1 comprising Allig, Hamrata and Grenja, and NA2 comprising Nefred and Deglet Nour, respectively.

Finally, the analysis of chloroplast genomes not only allows to solve the deeper branches of plants phylogeny and reconstruct phylogeographic patterns, but also contributes to the development of more effective DNA barcode markers for molecular characterization of germplasm and food products [39], and to the screening of genetic resources for breeding and genetic engineering [40].

This work includes the first massive resequencing of both organelle genomes of date palm accessions from different cultivars collected from three Tunisian continental oases, which represent the most important Tunisia’s date palm production sites [34, 41]. Targeted bioinformatics pipelines were used to identify haplotypes, genome variants and annotation and to study patterns of evolutionary relationship. Finally, functional annotation of genomic variants was carried out *in silico* to estimate the SNPs effects on genes functions. Overall, the genetic resources developed in this research could be used for further phylogeographic and population genetics studies of date palms in different geographical areas. The long-term goal is to contribute to the creation of a valuable platform for assisting genetic breeding and industrial exploitation of this economically important monocot tree.

## Results

Out of the 171 samples analyzed, 135 represent female plants and 36 represent males (Table S1). Among females, only 68 accessions (~ 50%) were successfully attributed to a certain cultivar based on fruit traits. In particular, the cultivars Ammary, Hissa and Gosbi are characterized by early-ripening soft fruits (ripening period July 15th - August 15th ), while Besser Helou, Kintichi and Kenta produce dry fruits with mid-season (August 15th – September 30th ) or late (after September 30th ) maturation. Deglet Nour is characterized by semi soft fruits and late maturation, which historically took place in December in the Tunisian oases but has been brought forward to October in the last 20 years due to rising temperatures [42]. Each accession was identified as a different cultivar, with the following exceptions: six samples attributed to the cultivar Alig, three samples identified as Besser Helou, two samples as Deglet Nour, three samples as Ghars Souf, two samples as Gondi, 11 samples as Gosbi and two samples identified as Khwat Alig, for a total of 42 different cultivars (Table S1).

### Chloroplast and mitochondrial genomic haplotype analysis

We generated ~ 658 Gb of raw data with ~ 4.4 billion reads. After filtering low quality bases, ~ 652 Gb of clean data were obtained, with a clean base rate of 99.02% (Table S1). We obtained an average sequence depth of 761x for chloroplast genomes and 665x for mitochondrial genomes and no sample resulted to have less than 96x of average depth across the two organelle genomes. After filtering and cleaning, we identified a total of 201 SNPs in the 171 individuals, 45 SNPs in the chloroplast genome and 156 in the mitochondrial genome (Fig. S1). This set of markers and individuals was used in subsequent analyses.

**Fig. 1:**
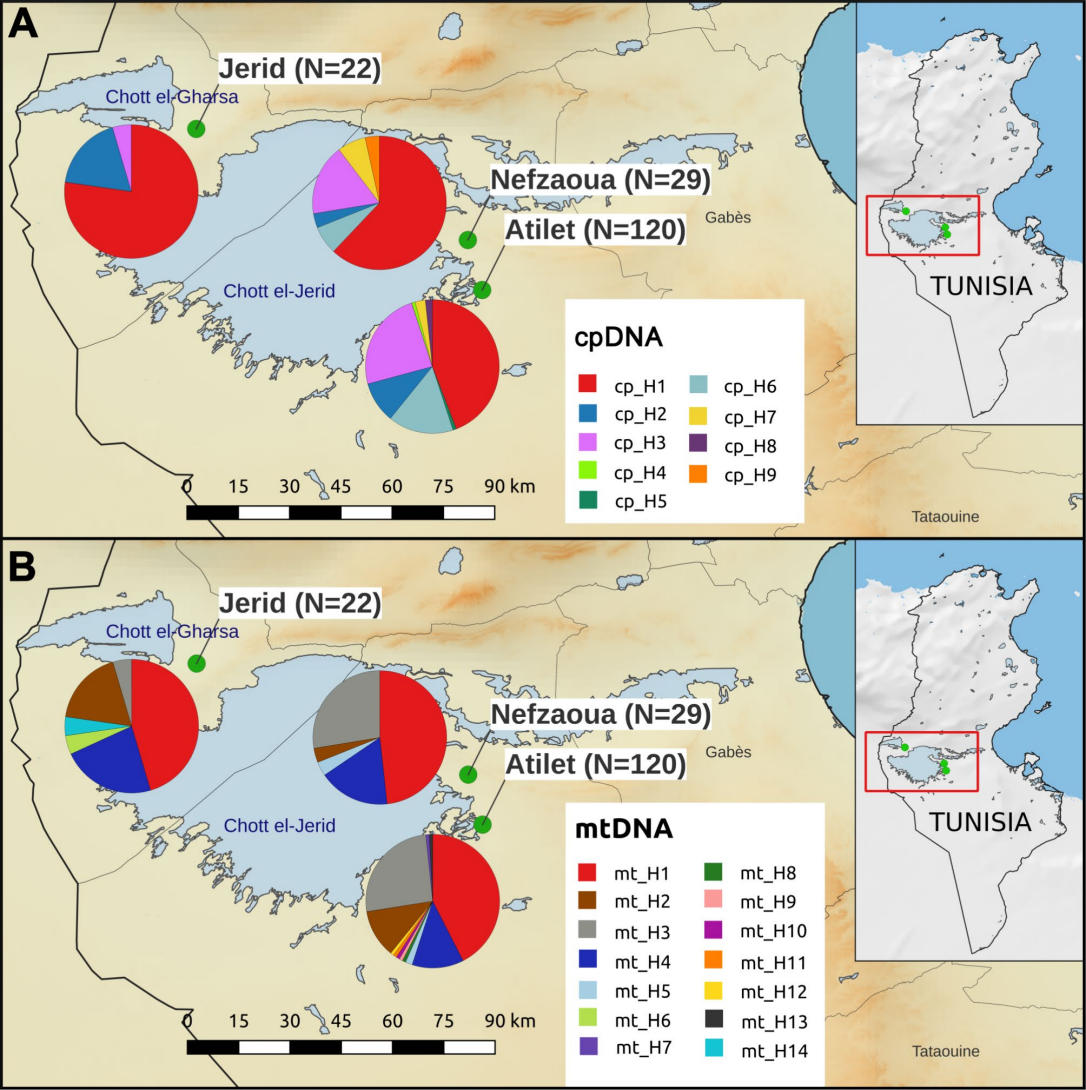
Haplotypes map. Map of the three sampled Tunisian date palm oases (green dots), with the detected haplotype frequencies for both (**A**) chloroplast (cpDNA) and (**B**) mitochondrial (mtDNA) genomes. The number of samples from each site is reported in brackets

Analysis of SNPs from the chloroplast genome identified 9 haplotypes (Table 1; Fig. 1A): H1 included most of the samples (88, 51.46%), followed by H3 (35, 20.47%), H6 (21, 12.28%), H2 (17, 9.94%), H7 (5, 2.92%) and H8 (2, 1.17%); H4, H5 and H9 were found only in single samples. As regards the cultivars represented by multiple accessions, the correspondence between cultivar and haplotype is not always linear. For Deglet Nour, Ghars Souf, Gondi and Khwat Alig, all accessions of the same cultivar were actually attributed to the same haplotype (H3, H2, H1 and H1, respectively; Table S1). Conversely, out of the six accessions identified as Alig cultivar, four were attributed to H1 and two to H6. The three accessions identified as Besser Helou were attributed to three different haplotypes (H1, H3 and H6). Finally, out of the 11 Gosbi accessions, two were attributed to H6, and the others to H1.

**Table 1:** Haplotype and nucleotide diversity in chloroplast and mitochondrial genomes found in sampled individuals, grouped for the oasis of origin of the sample

Oasis	Number of individuals	Chloroplast				Mitochondrial			
		Haplotypes	Private haplotypes	Haplotype diversity	Nucleotide diversity	Haplotypes	Private haplotypes	Haplotype diversity	Nucleotide diversity
Atilet	120	8	3	0.7164	1.32 × 10^− 4^	12	7	0.7287	8.75 × 10^− 5^
Jerid	22	3	0	0.3853	1.01 × 10^− 4^	6	2	0.7359	6.83 × 10^− 5^
Nefzaoua	29	6	1	0.5936	1.20 × 10^− 4^	5	0	0.6823	7.92 × 10^− 5^
TOTAL	171	9				14			

A total of 14 haplotypes were identified from the analysis of mitochondrial genome polymorphisms (Table 1; Fig. 1B): H1 haplotype was the most represented, with 75 individuals, followed by H3, with 40 individuals, H4 with 25 individuals, H2 with 19, and H5 with 3; all other haplotypes were represented by only one individual. The Alig samples were attributed to three different haplotypes (H4, H8 and H11) and Besser Helou samples to H1 and H3, while all the Deglet Nour, Ghars Souf, Gondi, Gosbi and Khwat Alig samples were attributed to the same haplotypes (H3, H2, H1, H1 and H4, respectively; Table S1). Overall, 20 haplotypes were resolved by combining information from the two organelle compartments.

The group of accessions sampled in Atilet oasis (N = 120) showed the highest number of haplotypes and private haplotypes for both the chloroplast (8 and 3, respectively) and mitochondrial genomes (12 and 7, respectively; Table 1). Accessions sampled in Jerid (N = 22) showed 3 chloroplast haplotypes and 6 mitochondrial haplotypes, 2 of which were private. Nefzaoua samples (N = 29) showed 6 chloroplast haplotypes, 1 of which was private, and 5 mitochondrial haplotypes. The level of haplotype diversity related to the mitochondrial genome was high and of the same magnitude in all oases (Table 1), while for the chloroplast genome it was high in Atilet oasis (0.7164), intermediate in Nefzaoua (0.5936) and lower in Jerid (0.3853). The highest level of nucleotide diversity was observed in the chloroplast genome, ranging from 1.32 × 10^− 4^ (accessions from Atilet) to 1.01 × 10^− 4^ (Jerid); accessions from Jerid also showed the lowest level of nucleotide diversity (6.83 × 10^− 5^) for the mitochondrial genome, however in the same order of magnitude of the other two oases, Nefzaoua and Atilet (7.92 × 10^− 5^ and 8.75 × 10^− 5^, respectively).

**Fig. 2:**
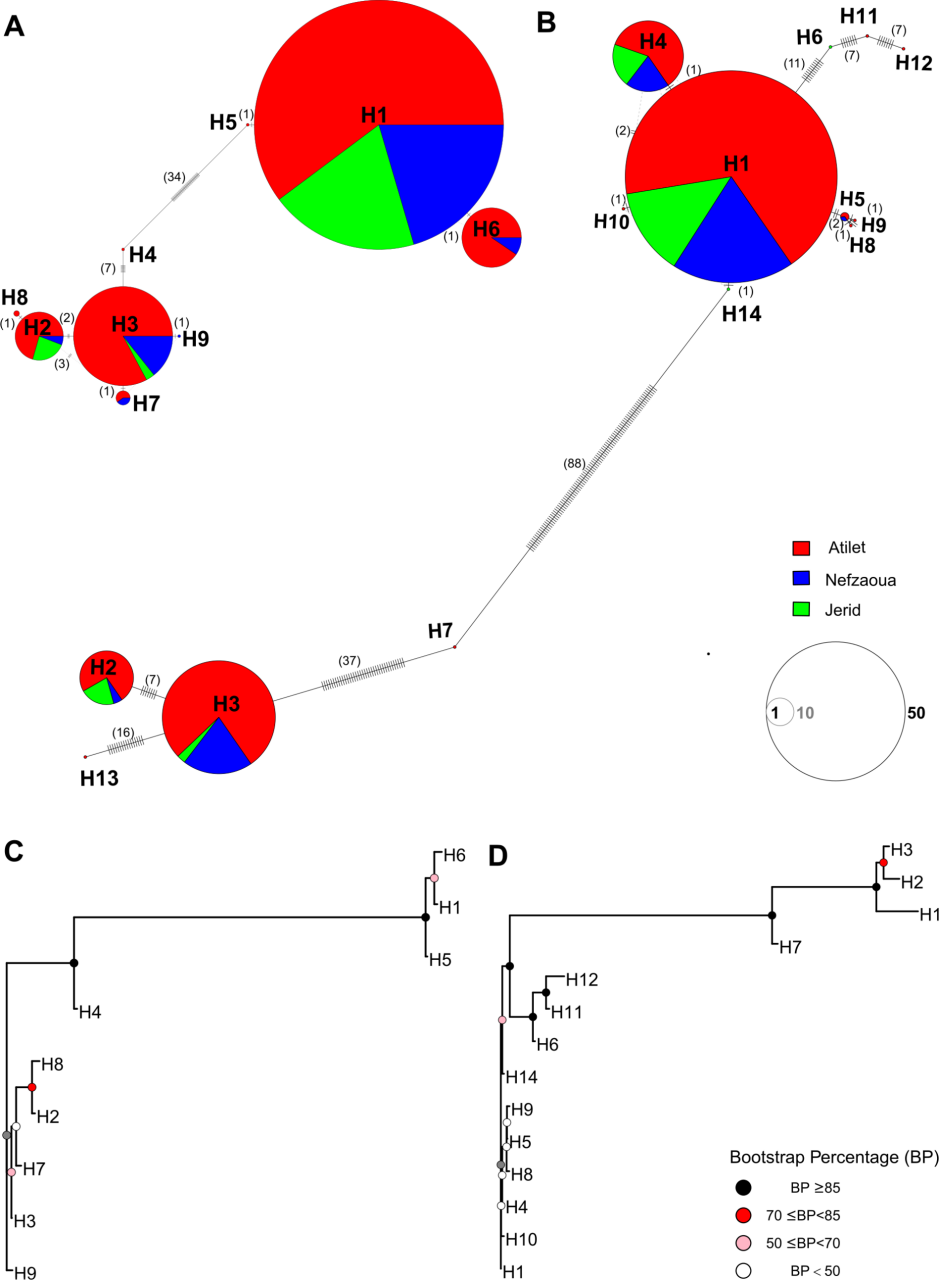
Haplotype networks and neighbor-joining trees. Chloroplast and mitochondrial haplotype networks (**A** and **B**, respectively) and neighbor-joining trees (**C** and **D**, respectively). In the haplotype network colors indicate the sampled oases and circle size is proportional to the number of individuals (circle sizes for 1, 10 and 50 individuals are shown in different shades of grey). Black bars on the branches and numbers in brackets indicate the number of SNPs between haplotypes

Analysis of the chloroplast and mitochondrial haplotype networks identified two main groups, in both cases centered on the H1 and H3 haplotypes, separated by several mutations, while the other, less frequent haplotypes were arranged as satellites around these two (Fig. 2A,B). The number of SNPs found between haplotypes is detailed in Table S2. Among haplotypes identified in the chloroplast genome, a maximum variation of 34 nucleotides was detected (between H4 and H5) and, similarly to the network, the neighbor-joining tree grouped the haplotypes in two clusters with high branch support (bootstrap value = 100%; Fig. 2C). Within haplogroup 1 (comprising H1, H5, H6), haplotypes are differentiated by 1 SNP; within haplogroup 2, a maximum variation of 7 nucleotides was detected between the remaining haplotypes (Fig. 2A; Table S2). Similarly, two highly supported clusters were observed in the neighbor-joining tree of the mitochondrial haplotypes (Fig. 2D), reflecting a maximum variation of 88 nucleotides (between H7 and H14). Within each mitochondrial haplogroup, a maximum variation of 37 nucleotides was detected; again, there are haplotypes distinguished by only one nucleotide, such as the pairs H5-H8, H5-H9, H1-H10, H1-H14, H1-H4 (Fig. 2B; Table S2).

**Fig. 3:**
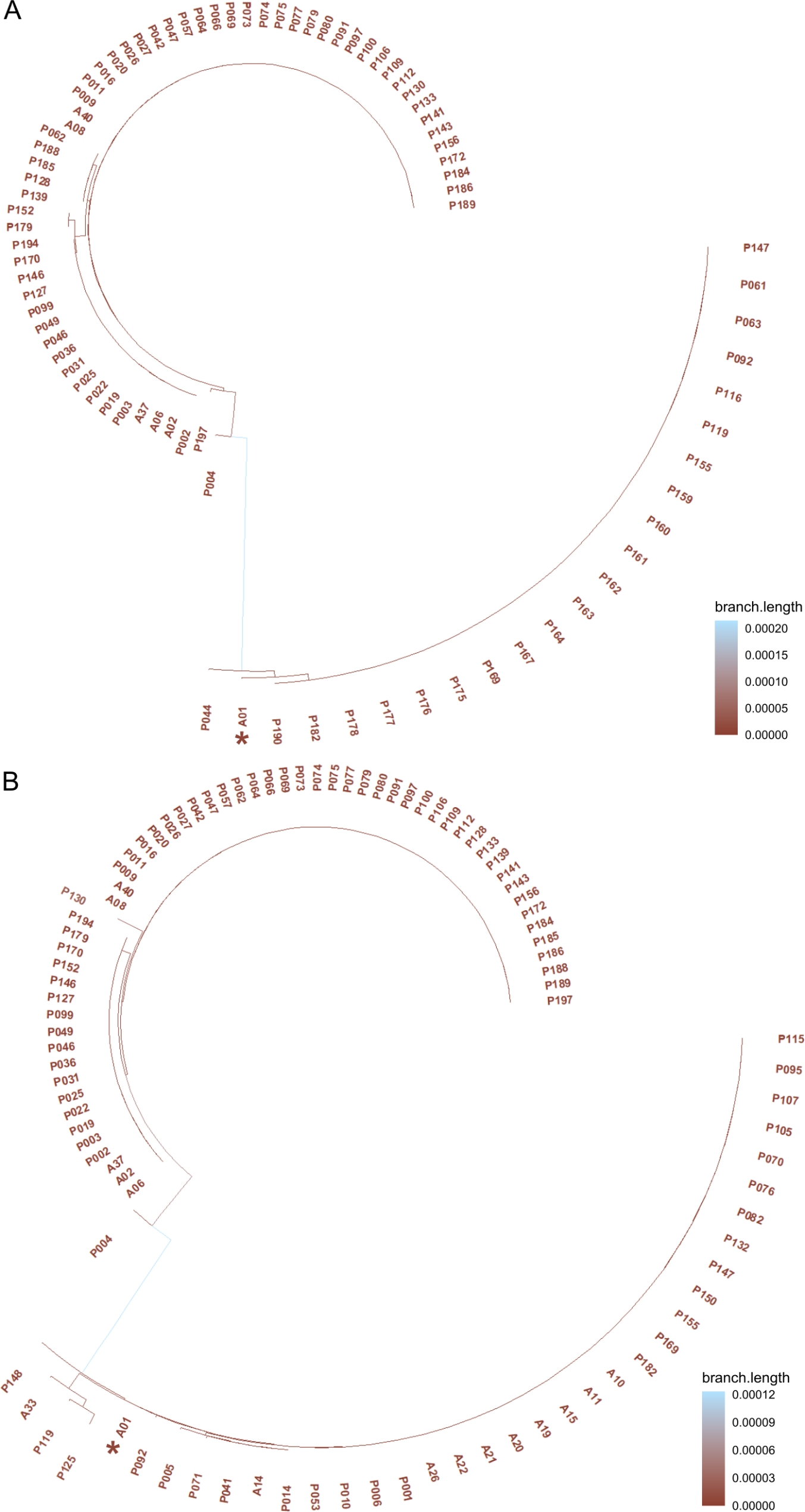
Phylogenetic trees. Phylogenetic tree of date palm samples based on chloroplast (**A**) and mithocondrial (**B**) sequences, built with PEGAS following Toparslan et al. [43]. To improve readability, the entire H1 haplotype is represented by sample A01 only, highlighted by an asterisk. Both the analyses showed the existence of two main phylogroups: one larger and homogeneous, the other smaller and more variable

In agreement with previous analysis, the phylogenetic relationships between organelle haplotypes led to the clustering of the 171 date palm samples in two phylogroups (Fig. 3A,B; Fig. S2). The larger group showed more homogeneity with no or very short internal branching, while the smaller group showed more pronounced internal subgroups. The two phylogroups corresponded to the two main groups found in the haplotype networks (Fig. S2). Both haplogroups were well represented in the three oases, although haplogroup 2 was always lower in frequency: it comprised the 23%, the 31% and the 39% of the samples in Jerid, Nefzaoua and Atilet, respectively (Fig. S3).

Finally, the analysis of plastidial SNPs revealed an exact match in alleles and positions along the sequence between haplotypes H1 and H3 and haplotypes NA1 and NA2 already identified by Mohamoud et al. [3] (Fig. S1). As regards mitochondrial sequences, the high number of indels did not allow a direct comparison.

### Functional annotation of variants

**Fig. 4:**
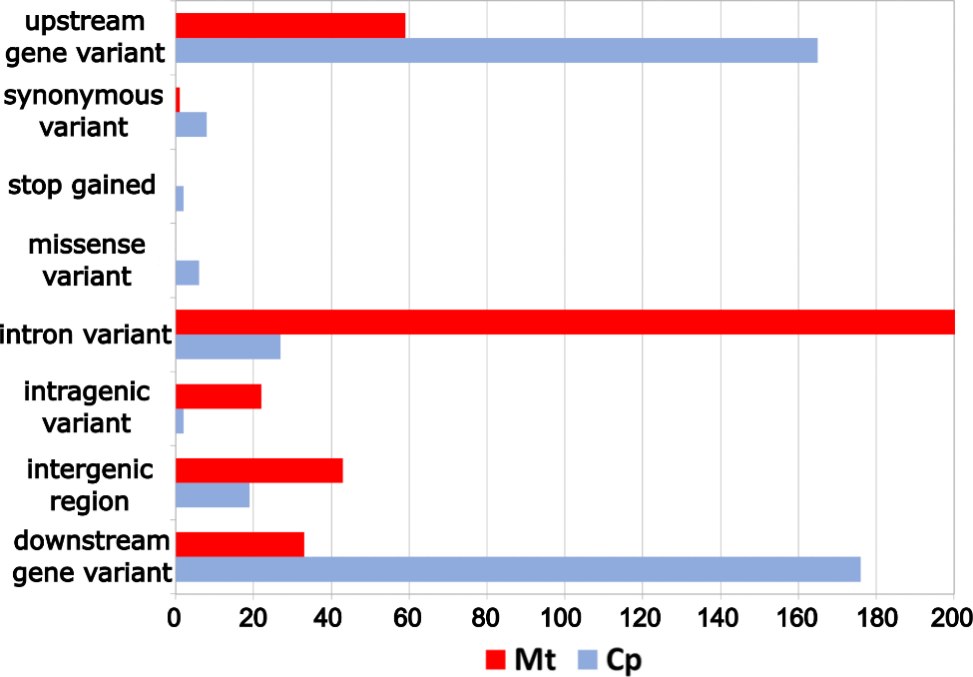
SNP classes. SNPs classification according to their effect and distribution across date palm chloroplast (Cp) and mitochondrial (Mt) genomes. The *x* axis displays the number of predicted effects, and the *y* axis the different effects specified using the sequence ontology terms

The mitochondrial genome had a variant rate of 1 in every 4583 bases. Upon annotation of 156 mt SNPs, we found a total of 371 effects (Fig. 4), as individual variants can exhibit more than one effect. Based on their impact, the analysis revealed that only 0.3% of SNPs have a low impact (i.e., are generally harmless or unlikely to modify protein behavior), while the great majority were non-coding variants or variants affecting non-coding genes. The gene with the highest number of variants having a modifier impact is nad2. The distribution of SNPs in the mitochondrial genome revealed a higher concentration in the intron regions (57% of SNPs were classified as intron variants). The effects of the remaining SNPs portion were identified as upstream, intergenic, intragenic, and downstream gene variants. Only one mutation was found in the exon region and produced a synonymous variant in an RNA polymerase gene.

There were 405 potential effects produced by the 45 chloroplast SNPs. The plastome had a SNPs density of 1 variant every 3521 bases. Consistent with the mt genome, the main class of impact identified was modifiers, but small percentages of SNPs with moderate, low, and high impact (1.5%, 2% and 0.5%, respectively) were also detected in the cp. genome. Concerning the effect types of SNPs, the results showed that most of the genomic variations were located in the upstream and downstream regions of the genes. A small percentage of SNPs (4%) was found in the exon regions, producing mainly synonymous and missense variants (causing an amino acid substitution). These variants, deemed to be of moderate impact, occurred in 4 genes: ycf1, rpl16, rpoC1 and rpoC2 genes. Among these, the ycf1 gene appears to be the most affected, as it contains three missense SNPs. Furthermore, SNPs with a stop-gain effect (0.5%), which have a disruptive impact on the protein, were identified in the genomic variants of cp., involving the petD and rpl16 genes. Finally, the Ts/Tv ratio was 0.11 for the mitochondrial genome and 1.24 for the chloroplast genome. To verify this statistic, we calculated the organellar Ts/Tv ratio for date palm with data from Mohamoud et al. [3]. We obtained a difference between Ts/Tv of cp. and mt of the same magnitude as our data: mitochondrial Ts/Tv = 0.03 and plastidial Ts/Tv = 1.6.

## Discussion

As in other North African regions [43], the Tunisian oases system is characterized by a large number of cultivars that derive from selection operated within the oases, aiming to optimize fruit production and quality under different environmental contexts. Identification of date palm cultivars based on a morphological approach is muddled by the high adaptive flexibility of the species, and relies on the expertise and the traditional knowledge of local growers [44]. This is further complicated by the many names that characterize differences related to fruit quality and morphology. Moreover, the lack of variety information for male individuals and consequently the reliance on the local expertise for the production of quality dates through selective crosses, makes these techniques non-reproducible and the results from different sites non comparable, also increasing the risk of errors and loss of both genetic and cultural heritage. This study is among the first exploring extensively the Tunisian date palm germplasm, based on whole chloroplast and mitochondrial whole-genome sequencing as a reliable method to assess genetic variation of date palm. The lack of a univocal correspondence between cultivars identified using phenotypic traits and haplotypes is evident from our results, with multiple accessions for the same cultivar attributed to different haplotypes. Moreover, these results do not seem to be related to the sampling location: considering for example the accessions identified as Gosbi cultivar, some samples from the three different oases were attributed to the same haplotype H1 according to cp. sequences, while samples from the same oasis Atilet were attributed to both haplotypes H1 and H6 (Table S1).

We found that the chloroplast genome is slightly more variable than the mitochondrial genome in terms of SNPs density. Annotation of the identified SNPs in both organellar genomes showed that almost all mutations fall in non-coding regions, as expected due to lower sequence conservation and reduced selection pressure in non-coding regulatory regions compared to coding regions. In mitochondria, most SNPs were identified in intergenic regions, whereas in the cp. genome, SNPs were much more abundant in the upstream and downstream regulatory regions of genes. However, some mutations have been identified in the cp. genome that could have a high or moderate impact on gene function. This small percentage of SNPs with a relevant effect on genes (4%) could be useful for developing DNA markers. Further studies on these polymorphisms could open up prospects for marker-assisted selection, taking into account nuclear-cytoplasmic interactions for improving date palm cultivars and studying adaptation.

The striking difference in the Ts/Tv ratio between cp. and mt genomes in the date palm was confirmed by the same analysis performed with another published date palm dataset [3]. A difference of the same magnitude, although of opposite direction, was observed in soybean [45] and Ginkgo [46] where Ts/Tv were 0.99 and 2.29 in mtDNA and 0.2 and 0.61 in cpDNA, respectively. It has already been reported that the rate of transversions can be slightly higher than that of transitions in the date palm mitochondrial genome [18]. The unusual transition transversion ratio was already observed in other species [47] and again in *Phoenix dactylifera* [38] where ~ 70% of the mitochondrial substitutions were transversions, probably due to the overwhelming ratio of intergenic regions.

The number of haplotypes observed in our study is small compared to other work on Tunisian cultivars done with specific loci e.g., of the chloroplast genome [27, 28]. Indeed, the applied method is highly conservative, eliminating indel-type and microsatellite polymorphisms, but it is however required to reduce the risks of intra-individual polymorphisms due to the potential phenomena of chloroplastic (nupt) and mitochondrial (numt) DNA transfer to the nucleus [48].

**Fig. 5:**
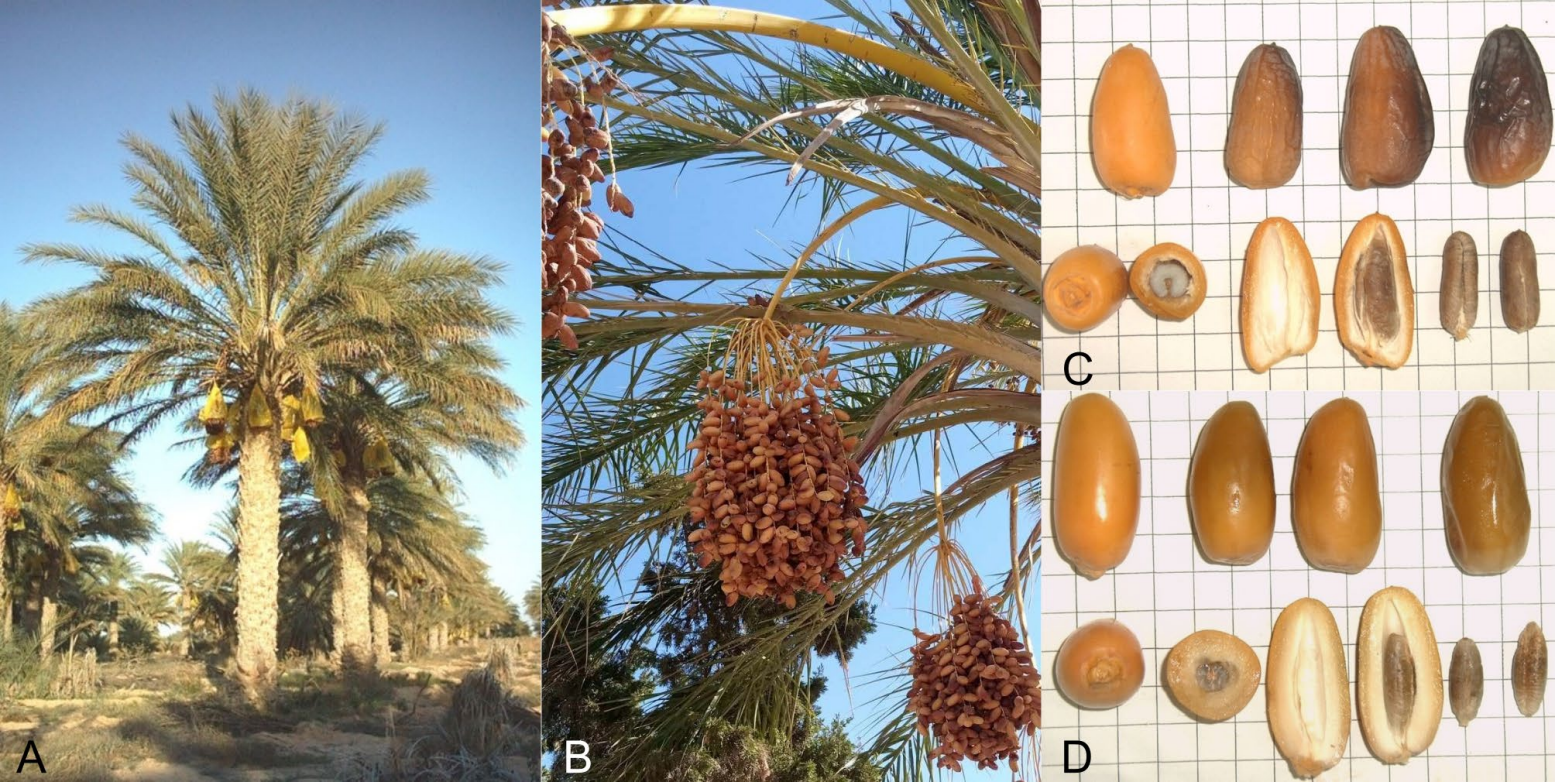
*Phoenix dactylifera*. Deglet Nour tree (**A**) and detail of the fruits (**B**). Morphological differences between Hamra (**C**) and Deglet Nour (**D**) dates

The most frequent haplotype in the Tunisian cultivars was H1, considering both cp. and mt genomes: together with its (less frequent) satellite haplotypes, H1 also constituted the main haplogroup in terms of haplotype frequencies. The second most abundant cp. and mt haplotype was H3, which is close to the haplotypes characteristic of the eastern date palm growing area [3] and significantly divergent from H1, as shown by the haplotype networks and phylogenetic trees (Figs. 5 and 2). It is worth noting that the polymorphic positions identified in our study correspond exactly to those defining the two North-African main haplotypes identified in the study by Mohamoud et al. [3], with our cp. H1 and H3 haplotypes corresponding to their NA1 and NA2 haplotypes, respectively (Fig. S1). In particular, among the Tunisian cultivars considered, only Alig, Deglet Nour and possibly Hamra (indicated as “Hamrata” by Mohamoud et al. [3]) are present in both the studies. Alig and Hamra(ta) cultivars were grouped into the NA1 haplotype by Mohamoud et al. [3], and into H1 in the present study (with the only exception of samples P119 and P155, attributed to H6), while Deglet Nour was attributed to a significantly different haplotype (NA2 and H3, respectively). In addition, having analyzed more accessions, we found additional haplotypes that are phylogenetically closely related (i.e. separated only by a few mutations) to the main two, from which they probably originated.

Most female accessions (96/135) belong to the H1 haplogroup, supporting the hypothesis that Tunisian date palm production relies on a relatively homogenous group, in terms of organellar genomic variation (Fig. 3, Table S1). The membership of Deglet Nour to the haplogroup centered in H3 supports the hypothesis of [49] that this cultivar was introduced to the Jerid oasis in the 16th century from Algeria. Moreover, our results on the divergence of Deglet Nour from the haplogroup 1 (including the most frequent haplotype) agree with both Zehdi-Azouzi et al. [36] and Mohamoud et al. [3] that Deglet Nour is genetically closer to the Arabian Gulf haplotype than to the major North African haplotypes. However, most of the male trees belong to H3, along with the Deglet Nour and Besser Helou cultivars. This suggests that the male trees may have been developed from the elite cultivar Deglet Nour (the most ubiquitous cultivar in Tunisia). In fact, Tunisian agricultures do not have a recognized male cultivar and obtain their pollinators from germination of seeds [50] of unknown cultivars. So, it is very likely that seeds of Deglet Nour fruits were the origin of the majority of male trees in Tunisia. In Tunisia, date palm has been cultivated for more than 3,000 years [51]. The domestication center of Tunisian date palm is generally believed to be located in the south-west of the country, next to the Berber and Roman settlements. Then, the distribution and selection of cultivars in different geographic regions were affected by the local environmental conditions such as climate and soil [52]. The date palm propagation patterns gave rise to and maintained a peculiar genetic diversity, and our finding based on whole chloroplast and mitochondrial genomes support this scenario.

The date palm gene pool observed in southwestern Tunisia and the information provided by our annotated organellar genome offer valuable resources for the breeding and genetic improvement of this important fruit tree crop. Indeed, organellar genome sequencing allowed the genetic characterization of both male accessions, whose variety is usually disregarded by growers, but contribute significantly in the offspring phenotype, and female accessions whose cultivar that could not be identified in the present study through phenotypic traits. This study is therefore a valuable contribution to the genetic improvement of date palm cultivation through selective cross-breeding.

## Conclusion

In this study, we performed an extensive genetic survey of Tunisian date palm accessions by scanning polymorphisms of the complete chloroplast and mitochondrial genomes. Our results unveiled a previously ignored haplotype richness and framed the evolutionary relationships of Tunisian date palm organellar genetic resources within the date palm growing area. Phylogenomics analyses strongly supported two well-separated haplogroups corresponding to the Western including North African and the Eastern range of date palm cultivation. The polymorphisms highlighted in this work would contribute to further studies on genetic diversity and phylogeography of date palm and would help fine-tune DNA barcodes in *Phoenix* species. However, although the abundant genetic resources developed in this work would be valuable for genetic breeding programs and germplasm exploitation of this economically important species, an additional effort is needed at the nuclear genome level to fully explore date palm genetic diversity.

## Methods

### Plant material collection and DNA extraction

In the present study, sequences from 171 date palm accessions were produced and analyzed. We sampled individual leaves from the main Tunisian continental oases, Jerid and Nefzaoua, and from Atilet, a conservation plot located in the Institute of Arid Regions (IRA, Kébili, Tunisia) which maintains endangered cultivars and is therefore representative for the genetic diversity of Tunisian date palm [44] (Fig. 1). Cultivar identification for female plants, obtained by clonal multiplication, was determined by experienced taxonomists and according to fruit traits (e.g. fruit color and consistency, ripening period) as described by Ferchichi and Hamza [53] (Fig. 5). No cultivar name was assigned to male trees, being derived from seed germination. All voucher specimens with individual labels (“Sample ID” in Table S1) were deposited and are stored permanently in the Laboratory of Dry Land Farming and Oasis Cropping, Institute of Arid Regions (IRA, Kébili, Tunisia). One sample per individual was processed for total genomic DNA extraction, using the Invisorb Spin Plant Mini Kit (Invitek, Berlin, Germany). DNA purity was checked using agarose gel electrophoresis, while DNA concentration was measured using Qubit® DNA Assay Kit in Qubit® 2.0 Fluorometer (Life Technologies, CA, USA). High-quality genomic DNA was stored at -20^◦^C until sequencing.

### Sequence analysis: data cleaning, SNP calling

We used a genome skimming approach, sequencing the whole genomic DNA in paired-end mode (2 × 150 bp reads) at low nuclear genome coverage on an Illumina HiSeq 4000 platform. Library construction and sequencing were conducted by Novogene Co., Ltd. (Beijing, China). Sequencing reads were deposited in GenBank (BioProject ID: PRJNA951931). Raw reads were cleaned using Trimmomatic [54] with the following options: trailing: 10; leading: 10; sliding window: 4:20; seed mismatches: 2; palindrome clip threshold: 30; simple clip threshold: 10 and minlen: 40. Sequence analysis was performed following Scarcelli et al. [48]. The mapping step was performed using BWA MEM 0.7.17-r1188 [55] with the -M and -B 4 options against NCBI reference GU811709.2 for mapping plastidial reads and NC_016740.1 for mapping mitochondrial reads. Samtools 1.10 [55] with –B option was used to generate a mpileup file. We used Varscan 2.3.7 mpileup2snp [56] to call SNPs (options –min-var-freq of 0.5, a –min-freq-for-hom of 0.5, a minimum base quality of 30, and a min coverage of 5). The resulting Variant Call Format file (VCF) was filtered using VCFtools v0.1.16 [57] to exclude indels, missing site positions, heterozygote positions, and individuals with more than 20% of missing data; only biallelic sites with a minimum depth of 5 and a minimum genotype quality of 10 were kept. VCF files were then converted into fasta files for downstream analysis.

### Analysis of organellar genomes variation

Chloroplast and mitochondrial haplotypes were inferred with the package PEGAS [58, 59] in R 4.2.1 [60], following the workflow proposed by Toparslan et al. [61]. Genetic relatedness between haplotypes were estimated and visualized through minimum spanning networks. For both organellar genomes, the genetic structure of haplotype diversity and the maximum-likelihood phylogenetic trees of the inferred haplotypes were inferred using the R package *fastbaps* [62]. Finally, alleles in polymorphic sites for cp. and mt haplotypes were visualized converting the VCF file into PHYLIP formatted sequence using vcf2phylip v1.5 [63]. Results were then compared with haplotypes from Mohamoud et al. [3]. Maps of the geographic distribution of detected haplotypes and haplogroups were produced using QGIS v3.20 (http://www.qgis.org) and the publicly available NASADEM Digital Elevation Model [64] and Tunisia Inland Waters map [65].

### Annotation of SNPs

Functional annotation of chloroplast and mitochondrial genomic variants was performed using SnpEff (version 5.0e; [66]) using the sequence ontology. Since annotations for the date palm genomes are not available in the built-in SnpEff databases, custom annotation databases were created for each organelle, based on data available in GenBank (NC_016740 and GU811709, for mitochondria and chloroplast, respectively). Variants were annotated according to their effect (high, moderate, modifier and low), functional class (missense, nonsense, silent) and genomic region (downstream, upstream, exon, intron, transcript, intragenic and intergenic). Furthermore, DNA substitution mutations (transitions and transversions) ratio (Ts/Tv) as well as changes in amino acids were identified. We calculated the organellar (Ts/Tv) ratio also for date palm haplotypes reported by Mohamoud et al. [3] using SnpSift [67].

### Electronic supplementary material

Below is the link to the electronic supplementary material.

Supplementary Material 1

Supplementary Material 2

Supplementary Material 3

## Data Availability

The datasets generated during the current study are available in the NCBI repository under BioProject accession PRJNA951931 (https://www.ncbi.nlm.nih.gov/bioproject/PRJNA951931/).

## Abbreviations

H: Haplotype (Chloroplast and Mitochondria)
cp: Chloroplast
mt: Mitochondria
NCBI: National Center for Biotechnology Information
SNP: Single Nucleotide Polymorphism
VCF: Variant Call Format file
WGS: Whole Genome Sequencing
