# Supplementary material for: Whole mitochondrial and chloroplast genome sequencing of Tunisian date palm cultivars: diversity and evolutionary relationships
Source: BMC Genomics. 2023 Dec 13;24:772. doi: 10.1186/s12864-023-09872-7 (PMC10720229; doi:10.1186/s12864-023-09872-7)
Supplement: Supplementary file 2 — Supplementary Material 2 [file 12864_2023_9872_MOESM2_ESM.pdf]

Whole mitochondrial and chloroplast genome sequencing of Tunisian date palm cultivars: diversity and evolutionary relationships

Authors: Hammadi Hamza, Sara Villa, Sara Torre, Alexis Marchesini, Mohamed Ali Benabderrahim, Mokhtar Rejili & Federico Sebastiani

Table S2 Number of SNPs between plastidial (A) and mitochondrial (B) haplotypes.

A

	**H1**	**H2**	**H3**	**H4**	**H5**	**H6**	**H7**	**H8**	**H9**
**H1**	0	44	42	35	1	1	43	45	41
**H2**	44	0	2	9	43	45	3	1	3
**H3**	42	2	0	7	41	43	1	3	1
**H4**	35	9	7	0	34	36	8	10	8
**H5**	1	43	41	34	0	2	42	44	40
**H6**	1	45	43	36	2	0	43	44	42
**H7**	43	3	1	8	42	43	0	2	2
**H8**	45	1	3	10	44	44	2	0	4
**H9**	41	3	1	8	40	42	2	4	0

B

	**H1**	**H2**	**H3**	**H4**	**H5**	**H6**	**H7**	**H8**	**H9**	**H10**	**H11**	**H12**	**H13**	**H14**
**H1**	0	129	126	1	2	11	89	3	3	1	16	21	142	1
**H2**	129	0	7	130	131	140	40	132	132	130	145	150	23	128
**H3**	126	7	0	127	128	137	37	129	129	127	142	147	16	125
**H4**	1	130	127	0	3	12	90	2	4	2	15	22	143	2
**H5**	2	131	128	3	0	13	91	1	1	3	18	23	144	3
**H6**	11	140	137	12	13	0	100	14	14	12	7	10	131	12
**H7**	89	40	37	90	91	100	0	92	92	90	105	110	53	88
**H8**	3	132	129	2	1	14	92	0	2	4	17	24	145	4
**H9**	3	132	129	4	1	14	92	2	0	2	19	24	145	4
**H10**	1	130	127	2	3	12	90	4	2	0	17	22	143	2
**H11**	16	145	142	15	18	7	105	17	19	17	0	7	138	17
**H12**	21	150	147	22	23	10	110	24	24	22	7	0	131	22
**H13**	142	23	16	143	144	131	53	145	145	143	138	131	0	141
**H14**	1	128	125	2	3	12	88	4	4	2	17	22	141	0
