# Supplementary material for: Whole mitochondrial and chloroplast genome sequencing of Tunisian date palm cultivars: diversity and evolutionary relationships
Source: BMC Genomics. 2023 Dec 13;24:772. doi: 10.1186/s12864-023-09872-7 (PMC10720229; doi:10.1186/s12864-023-09872-7)
Supplement: Supplementary file 3 — Supplementary Material 3 [file 12864_2023_9872_MOESM3_ESM.pdf]

Whole mitochondrial and chloroplast genome sequencing of Tunisian date palm cultivars: diversity and evolutionary relationships

Hammadi Hamza, Sara Villa, Sara Torre, Alexis Marchesini, Mohamed Ali Benabderrahim, Mokhtar Rejili & Federico Sebastiani

Supplementary figures

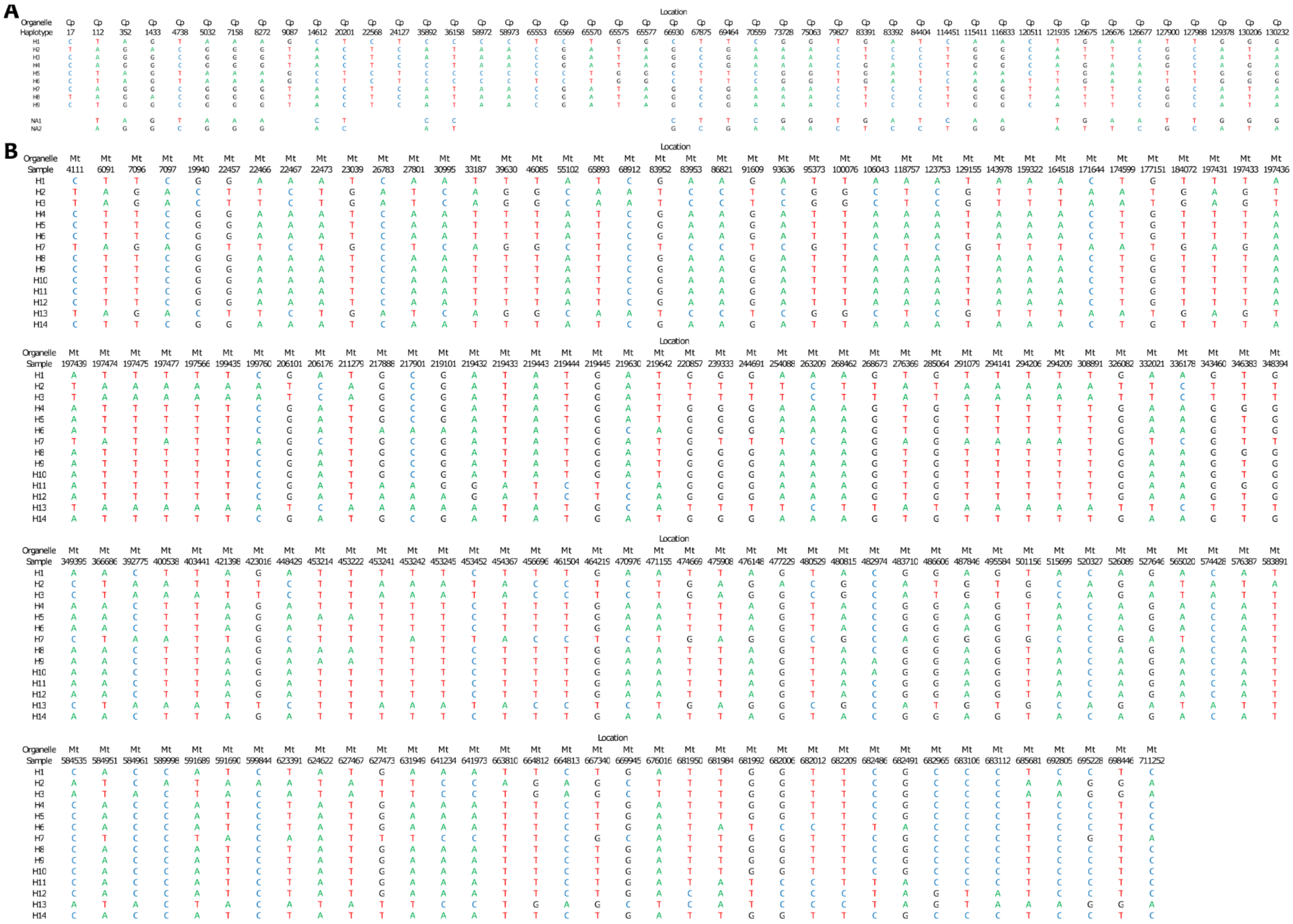

Fig. S1. Alleles in SNP sites. Alleles in SNP sites within date palm organelle genome sequences, grouped according to the main detected haplotypes. Locations correspond to base pair coordinates within chloroplast (Cp; A) and mitochondrial (Mt; B) genomes. For chloroplast, alleles found by Mohamoud et al. (2019) in haplotypes NA1 and NA2, corresponding to tunisian H1 and H3, are reported. Alleles and SNPs position were visualized with vcf2phylip v1.5 (Ortiz, 2018).

A

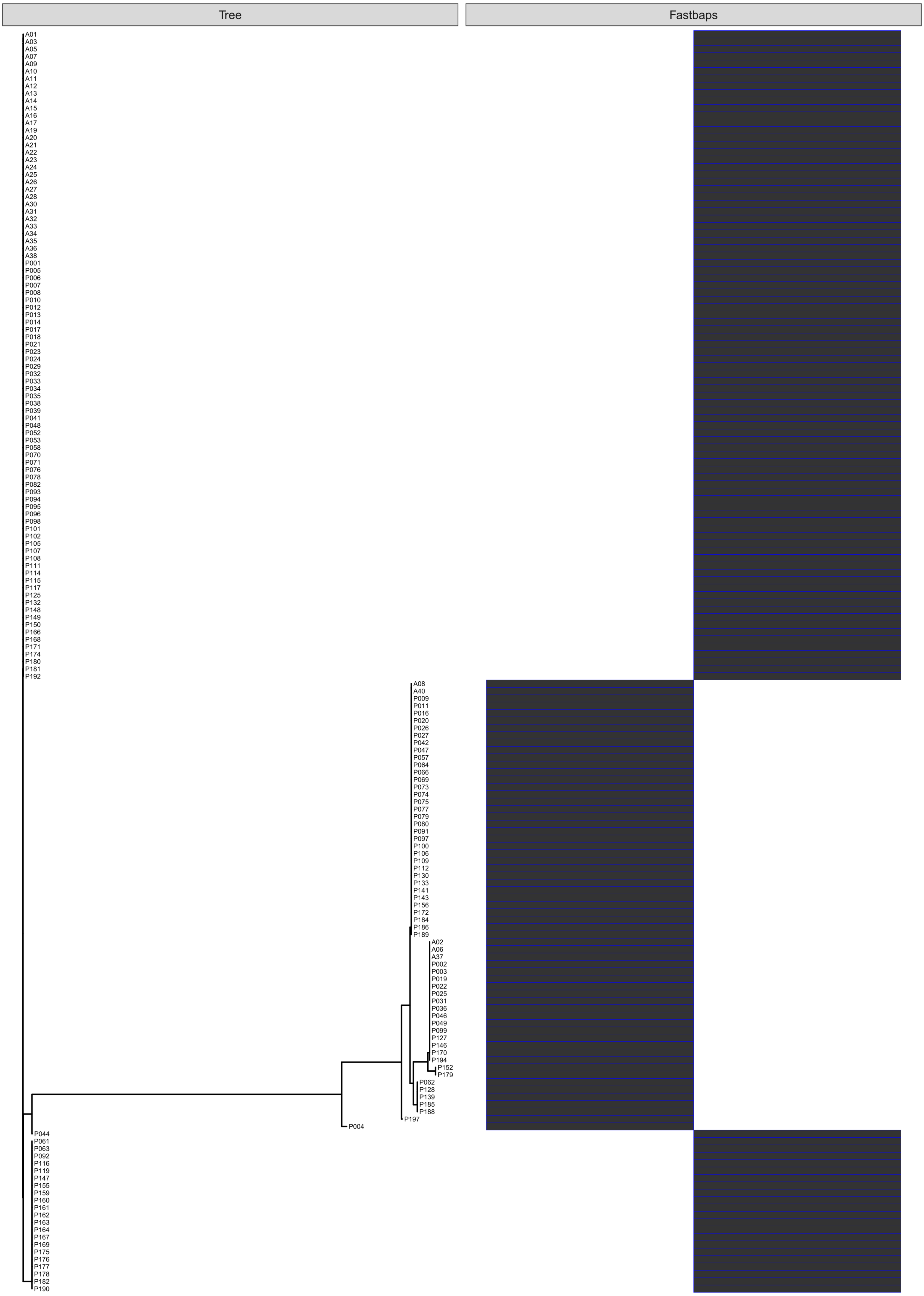
Tree
Fastbaps
A01
A03
A05
A07
A09
A10
A11
A12
A13
A14
A15
A16
A17
A19
A20
A21
A22
A23
A24
A25
A26
A27
A28
A30
A31
A32
A33
A34
A35
A36
A38
P001
P005
P006
P007
P008
P010
P012
P013
P014
P017
P018
P021
P023
P024
P029
P032
P033
P034
P035
P038
P039
P041
P048
P052
P053
P058
P070
P071
P076
P078
P082
P093
P094
P095
P096
P098
P101
P102
P105
P107
P108
P111
P114
P115
P117
P125
P132
P148
P149
P150
P166
P168
P171
P174
P180
P181
P192
A08
A40
P009
P011
P016
P020
P026
P027
P042
P047
P057
P064
P066
P069
P073
P074
P075
P077
P079
P080
P091
P097
P100
P106
P109
P112
P130
P133
P141
P143
P156
P172
P184
P186
P189
A02
A06
A37
P002
P003
P019
P022
P025
P031
P036
P046
P049
P099
P127
P146
P170
P194
P152
P179
P062
P128
P139
P185
P188
P197
P004
P044
P061
P063
P092
P116
P119
P147
P155
P159
P160
P161
P162
P163
P164
P167
P169
P175
P176
P177
P178
P182
P190

B

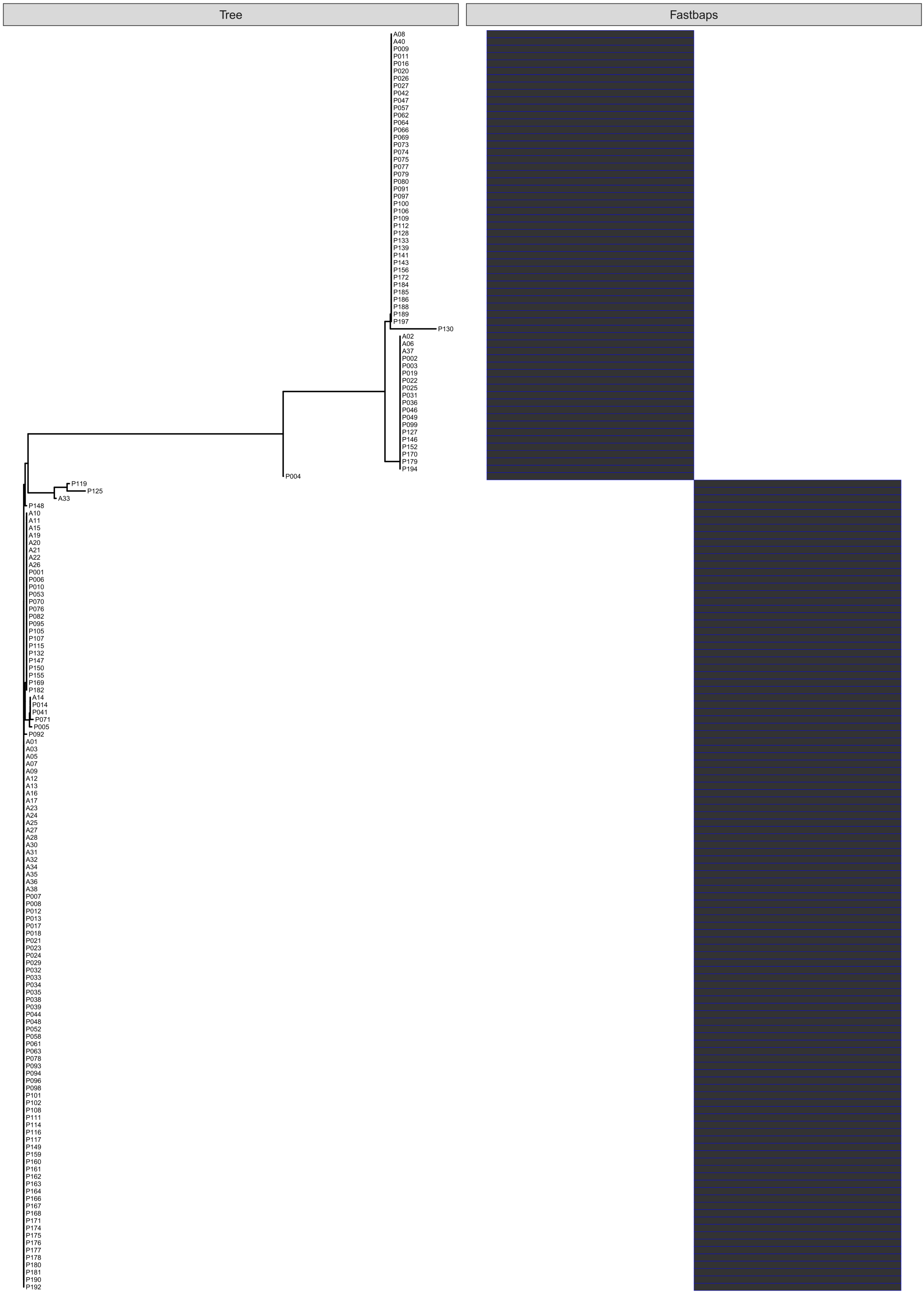

Tree
Fastbaps
A08
A40
P009
P011
P016
P020
P026
P027
P042
P047
P057
P062
P064
P066
P069
P073
P074
P075
P077
P079
P080
P091
P097
P100
P106
P109
P112
P128
P133
P139
P141
P143
P156
P172
P184
P185
P186
P188
P189
P197
P130
A02
A06
A37
P002
P003
P019
P022
P025
P031
P036
P046
P049
P099
P127
P146
P152
P170
P179
P194
P004
P119
P125
A33
P148
A10
A11
A15
A19
A20
A21
A22
A26
P001
P006
P010
P053
P070
P076
P082
P095
P105
P107
P115
P132
P147
P150
P155
P169
P182
A14
P014
P041
P071
P005
P092
A01
A03
A05
A07
A09
A12
A13
A16
A17
A23
A24
A25
A27
A28
A30
A31
A32
A34
A35
A36
A38
P007
P008
P012
P013
P017
P018
P021
P023
P024
P029
P032
P033
P034
P035
P038
P039
P044
P048
P052
P058
P061
P063
P078
P093
P094
P096
P098
P101
P102
P108
P111
P114
P116
P117
P149
P159
P160
P161
P162
P163
P164
P166
P167
P168
P171
P174
P175
P176
P177
P178
P180
P181
P190
P192

Fig. S2. **Phylogenetic tree and samples clustering**. Phylogenetic tree and clustering of date palm samples based on chloroplast (A) and mithocondrial (B) sequences, obtained with the maximum likelihood method using the R package *fastbaps* (Tonkin-Hill et al., 2019).

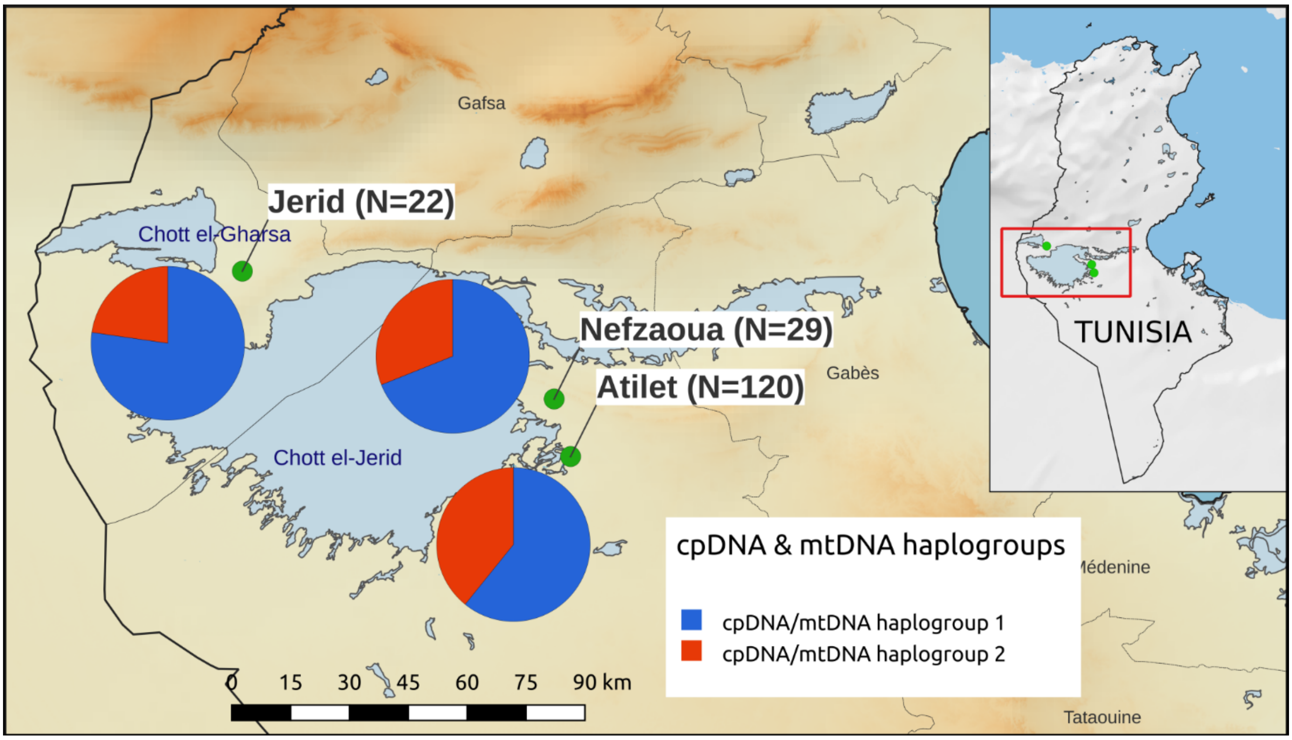

Fig. S3 Haplogroups map. Map of the three sampled Tunisian date palm oases, with the detected haplogroup frequencies for chloroplast (cpDNA) and mitochondrial (mtDNA) genomes.
